# Protective Effect of RNase on Unilateral Nephrectomy-Induced Postoperative Cognitive Dysfunction in Aged Mice

**DOI:** 10.1371/journal.pone.0134307

**Published:** 2015-07-30

**Authors:** Chan Chen, Jingjing Cai, Shu Zhang, Lu Gan, Yuanlin Dong, Tao Zhu, Gang Ma, Tao Li, Xiyang Zhang, Qian Li, Xu Cheng, Chaomeng Wu, Jing Yang, Yunxia Zuo, Jin Liu

**Affiliations:** 1 Department of Anesthesiology and Translational Neuroscience Center, West China Hospital, Sichuan University, Chengdu, Sichuan, China; 2 Department of Emergency Medicine, West China Hospital, Sichuan University, Chengdu, Sichuan, China; 3 Geriatric Anesthesia Research Unit, Department of Anesthesia, Critical Care and Pain Medicine, Massachusetts General Hospital and Harvard Medical School, MA, United States of America; Universidade de São Paulo, BRAZIL

## Abstract

Postoperative cognitive dysfunction (POCD) is a common complication after surgery, especially for elderly patients. Administration of RNase has been reported to exhibit neuroprotective effects in acute stroke. However, the potential role of RNase on POCD is unknown. Therefore, we sought to investigate whether RNase treatment could mitigate unilateral nephrectomy induced-cognitive deficit in aged mice. In the present study, twelve-month-old mice were administered RNase or an equal amount of normal saline perioperatively. All mice underwent Morris Water Maze (MWM) training 3 times per day for 7 days to acclimatize them to the water maze before surgical operation, and testing on days 1, 3 and 7 after surgery. We found that perioperative administration of RNase: 1) attenuated unilateral nephrectomy-induced cognitive impairment at day 3 after surgery; 2) reduced the hippocampal cytokines mRNA production and serum cytokines protein production at day 1 and day 7 (for MCP-1) after surgery, and; 3) inhibited hippocampal apoptosis as indicated by cleaved caspase-3 western blot and TUNEL staining at day 1 after surgery. In addition, a trend decrease of total serum RNA levels was detected in the RNase treated group after surgery compared with the untreated group. Further, our protocol of RNase administration had no impact on the arterial blood gas analysis right after surgery, kidney function and mortality rate at the observed days postoperatively. In conclusion, perioperative RNase treatment attenuated unilateral nephrectomy-induced cognitive impairment in aged mice.

## Introduction

Postoperative cognitive dysfunction (POCD) is a common complication after surgery, especially in elderly patients [1, 2]. It is known that POCD may occur in the acute and/or late stage following either cardiac or non-cardiac surgery, which is associated with poor health outcomes of the patients [3, 4]. The reported incidence of early POCD varies in different studies, with the highest around 80% in the first postoperative week [5], and lowest about 17% at one month after surgery [6]. Despite years of considerable progress towards understanding the molecular mechanisms involved in the development of POCD, the pathogenesis of POCD remains relatively unclear.

Increased studies have suggested that neuroinflammation may play a central role in the pathogenesis of POCD [7, 8]. However, clinical trials using anti-inflammatory drugs, such as non-steroidal anti-inflammatory drugs (NSAIDs), have failed to show either consistent beneficial effects in patients with mild cognitive impairment or usefulness in the treatment of established dementia [9]. There is growing evidence that systemic inflammation can induce exacerbation and progression of inflammatory responses or damage in the central nervous system (CNS) [10, 11]. Therefore, we reasoned that it might be of great importance to identify the inflammatory initiators that are involved in the mediation, amplification and perpetuation of postoperative neuroinflammatory reactions. Additionally, damping the systemic initiators at early stages may help to prevent the chain reaction that triggers the inflammatory or apoptotic response in the CNS.

Damage-associated molecular pattern molecules (DAMPs) are molecules might be generated and released during adverse conditions, such as tissue trauma, ischemia, and hypoxia [12]. DAMPs can trigger responses of immune system in the noninfectious inflammatory response. DAMPs encompass various types of molecules, (e.g., heat-shock proteins, high mobility group box 1 (HMGB1), RNA, and S100 proteins) [13]. Recent studies have demonstrated that HMGB1 and S-100β may be associated with POCD [14, 15]. Extracellular RNA (exRNA), released from necrotic cells, has been recently reported to be involved in pathological conditions such as myocardial ischemia/reperfusion (I/R) injury [16], atherosclerotic plaque formation [17], and focal cerebral ischemia [18]. Besides its role as the information bridge between DNA and protein, exposure of various cells to exRNA also can result in inflammatory responses through upregulation of the pro-inflammatory mediators [16]. Previous studies have shown that administration of Ribonuclease (RNase), the counterpart of exRNA, significantly reduced myocardial infarction in the mouse model of myocardial I/R injury. It is suggested that reduction of inflammation and apoptosis could be the possible molecular mechanism for the protective effects of RNase [16]. Notably, RNase has also been proven to be neuroprotective in a rat model of temporary middle cerebral artery occlusion, through reduction of leakiness of the blood brain barrier or brain edema formation [19]. Therefore, it is of great interest to test whether RNase treatment may play a role in POCD.

Therefore, in this study, we aimed to evaluate if RNase treatment may confer neuroprotection from POCD in a surgical model of aged mice. We also evaluated kidney function, performed arterial blood gas analysis after RNase administration, and further determined the possible mediators and the molecular mechanisms that may be associated with initiation and development of cognitive impairment.

## Materials and Methods

### Animals and Experimental Procedures

C57BL/6 male mice, aged 12 months, weighing 26–35g, were purchased from Sichuan University, P.R.China and maintained under controlled conditions on a 12 h light/dark cycle with free access to laboratory food and water. The study was approved by the Animal Care and Use Committee of Sichuan University (Permit Number: 20111020002), and conformed to the Guide for the Care and Use of Laboratory Animals published by the US National Institutes of Health (NIH Publication No. 85–23, revised 1996). All surgery was performed under the combination of ketamine and xylazine anesthesia, and all efforts were made to minimize suffering. Aged mice were randomly assigned to 3 groups: sham surgery plus placebo (S-S), surgery plus placebo (S-P), and surgery plus RNase (S-R). Briefly, mice were anesthetized by intraperitoneal injection of ketamine (120 mg/kg) and xylazine (4 mg/kg), a small transverse incision was made and the left kidney was removed (S-P and S-R groups), or only exposure of kidney was made without excision (S-S group). The S-R group received 3 doses of RNase A before, during and after the surgery respectively. The S-S and S-P groups only received normal saline of equal volume at corresponding time points. During the operation, body temperature of the mice was maintained at 36.5°C to 37.5°C. All the mice received 50μl of 0.2% ropivacaine subcutaneously for the post-operative analgesia.

### Morris Water Maze Test

**Fig 1:**
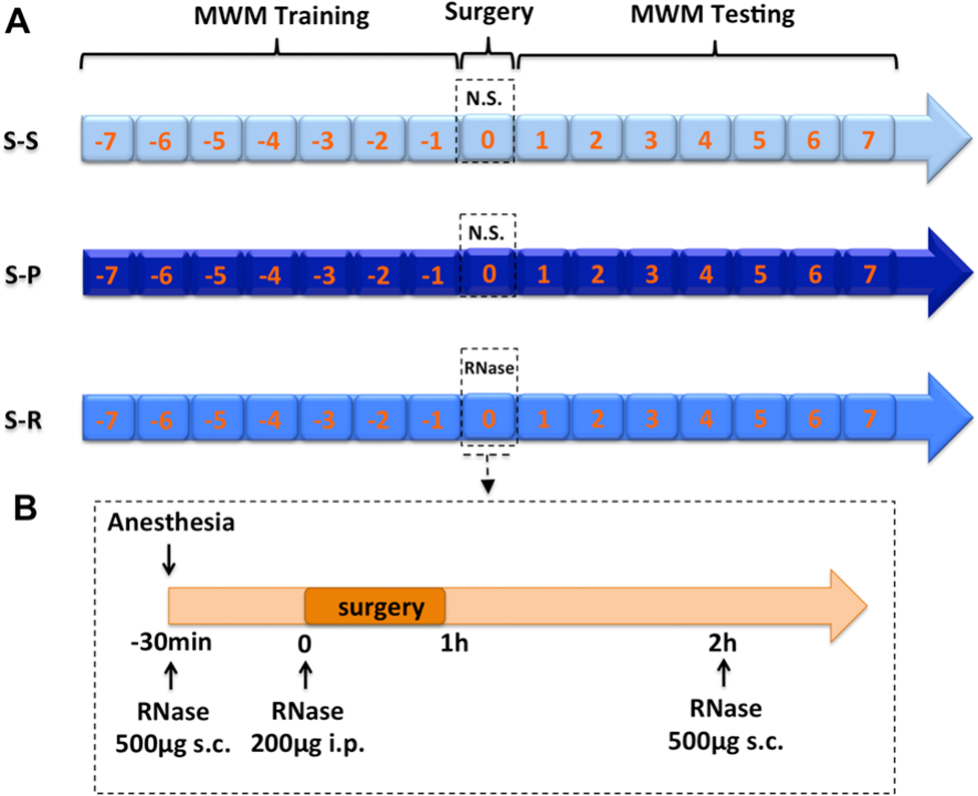
The schematic outline of the experimental protocol and the timeline of RNase administration. A, Schematic outline of the experimental protocol. B, Timeline of RNase A administration. MWM, Morris water maze; S-S, sham surgery plus placebo; S-P, surgery plus placebo; S-R, surgery plus RNase.

Morris Water Maze (MWM) was chosen for the assessment of learning and memory, one aspect of the cognition [20]. Briefly, the water maze consisted of a round tank (diameter: 120 cm, depth: 40 cm) of water rendered opaque by adding white non-toxic paint, and the water temperature was maintained at 25°C. A platform submersed 1 cm below surface was placed in one quadrant of the tank and maintained in the same position during all trials. Several visual clues were placed around the pool as the mice’s navigational reference points for locating the platform. A video camera in conjunction with the SMART video tracking software (Panlab, Barcelona, Spain) was used for the measurement of swimming speed, latency to escape to the platform, percentage of time spent in the target quadrant and distance swam to the platform. The training session consisted of 3 consecutive trials per day for 7 days before surgery (**Fig 1A**), during which the mice were placed in the desired start position in the maze facing the tank wall, and allowed to swim freely to the escape platform. Trial time limit is 90 seconds. If the mice did not find the platform within the time limit, they were gently guided to it. The testing session including 3 consecutive trials was performed for 7 days after surgery (**Fig 1A**). The probe tests with the platform removed were performed on the 3rd or 7th postoperative day. The probe test was 90 seconds in duration, and the percentage of time spent in the quadrant previously containing the submerged platform was recorded.

### In vivo RNase Administration

RNase A (Invitrogen), a bovine pancreatic ribonuclease with high efficiency for the degradation single-stranded RNA, was used in this study after filtration (30 kDa cut-off filter). Briefly, 3 doses of RNase A were administered as follows: 500 μg/100 μl, SC, 30 minutes prior to, 200 μg/200 μl, IP, right before, and 500 μg/100 μl, SC, 1 hour after unilateral nephrectomy (**Fig 1B**), which has been reported previously [16].

### Arterial Blood Gas Analysis

Immediately after the surgery, whole blood samples were taken with a heparin coated syringe from the left ventricles of mice from all the four groups (S-S, S-P, S-R, and naïve group, n = 3 per group) for arterial blood gas analysis. Blood gas analyzer (ABL800 FLEX, Radiometer Medical A/S, Copenhagen, Denmark) was used for the measurement of pH, blood gas (pCO2, pO2), electrolytes (cCl-, cCa++, cK+, cNa+), metabolites (cLac) and oximetry: ctHb, sO2.

### Serum Preparation

Mouse whole blood was collected in the RNase-free Eppendorf tubes at four time points: baseline, day 1, day 3 and day 7, after the cognitive tests. After collection of the whole blood, it was left undisturbed at room temperature for about 30 minutes, followed by centrifugation at 3,000 x g for 10 minutes at 4°C. The supernatant serum was collected. These experiments were performed in the strictly RNase-free conditions.

### Kidney Function Test

An automatic chemistry analyzer (BS-120 Chemistry Analyzer, Mindray) was used for the measurement of creatinine (CREA) and blood urea nitrogen (BUN), in the serum collected at the corresponding time points. Three mice aged 12 months maintained under the same controlled conditions as that of all the other 3 groups (S-S, S-P, and S-R) were used as the naïve control.

### Quantification of Total Serum RNA Concentration and Cytokines mRNA

Equal volume (100 μl) of serum from all the groups were used for the abstraction and quantification of total serum RNA. DNA-free total serum RNA was extracted by using Eastep Universal RNA Extraction Kit and quantified using Thermo Scientific NanoDrop 2000 spectrophotometer. These experiments were also conducted in strictly RNase-free conditions. Total RNA from hippocampus tissues was isolated using TRIzol reagent (Invitrogen Life Technologies). qRT-PCR was performed on a Mastercycler ep realplex realtime PCR system (Eppendorf, Hauppauge, NY). The level of GAPDH was used to normalize data in each individual sample. The primer sequence used for qRT-PCR were described previously [16].

### Quantification of Serum Cytokines Protein

Luminex multiplex fluorescent bead-based immunoassays (Milliplex MAP Mouse cytokine kit) were used to simultaneously detect multiple cytokine expression in the serum based on the manufacturer’s protocol. A set of selective cytokines was included in the Luminex assay to measure the cytokine protein expression levels in the serum samples.

### Western Blot Analysis

Total protein was extracted as described [21]. Equivalent amounts of proteins (100 μg) were separated by 10% or 15% SDS-PAGE and transferred onto a polyvinylidene fluoride (PVDF) membrane (Millipore). The membranes were sequentially incubated with anti-cleaved caspase-3 antibody (1:1000, Cell Signaling Technology, USA) overnight and anti-GAPDH antibody (1:2000, Cell Signaling Technology, USA) for 4 hours at 4°C. Immunoreactivity was detected with ECL fluorescent detection reagent, and analyzed by NIH Image J software. β-Actin was used as a loading control.

### TUNEL Staining

For detection of apoptosis in paraffin-embedded tissue, terminal deoxynucleotidyl transferase dUTP nick end labeling (TUNEL) staining was performed using an In Situ Cell Death Detection Kit (POD; Roche Diagnostics Corp., Indianapolis, IN, USA) following the protocol reported previously. Cells with brown nuclei were counted in 6 microscopic fields of the CA1 region (400 x) from each section of the hippocampus, and the percentage of TUNEL-positive nuclei was calculated.

### Statistical Analysis

All data were expressed as means ± SEM. The statistical analysis was performed using Graphpad Prism 6 software. Analysis of data was performed using one-way analysis of variance (ANOVA). Post hoc analysis (Student-Newman Keul’s test) was performed for multiple comparisons between different groups of these experiments. Probability values less than 0.05 (*P*<0.05) were considered statistically significant.

## Results

### RNase Treatment Attenuated Unilateral Nephrectomy-Induced Cognitive Dysfunction

**Fig 2:**
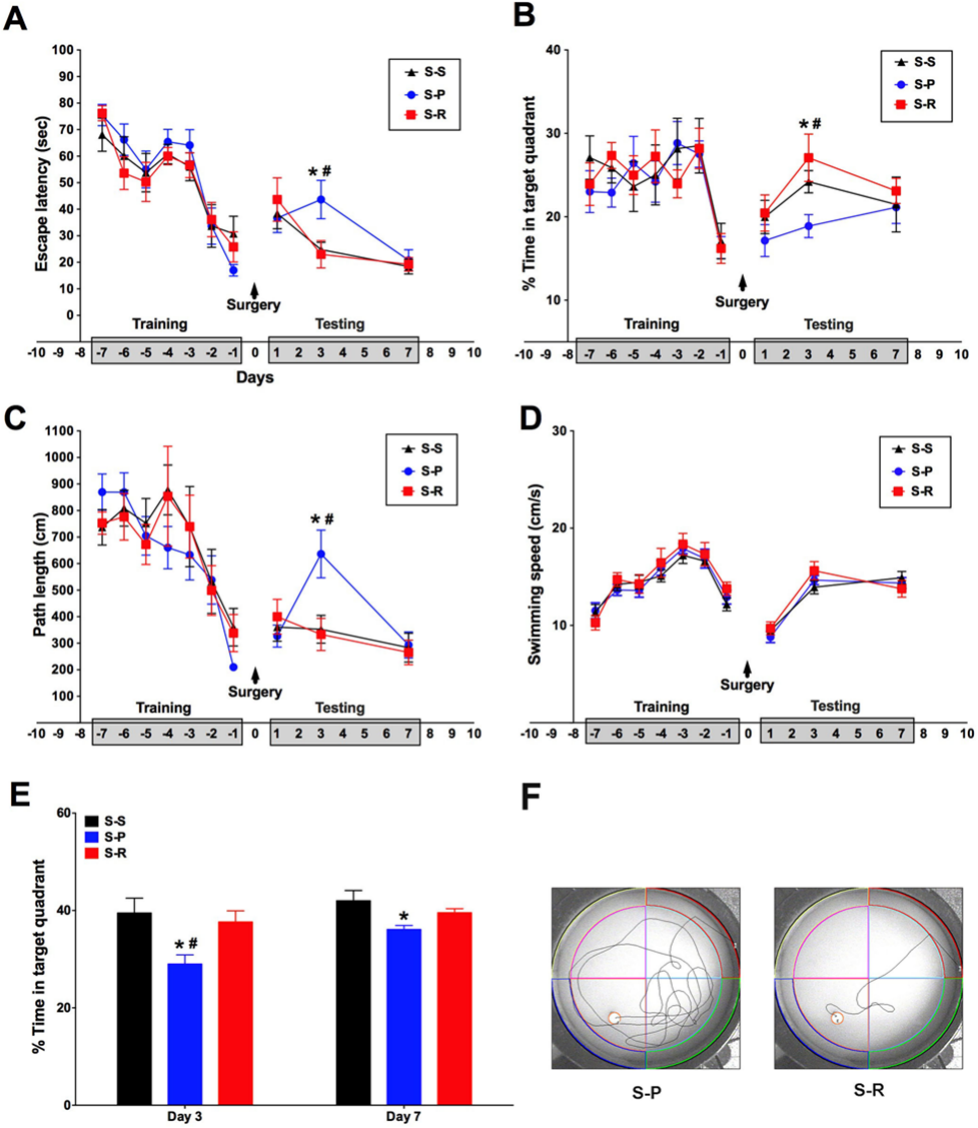
RNase treatment reduced cognitive impairment induced by unilateral nephrectomy surgery in aged mice. The MWM was used for the training of spatial memory formation in aged mice for 7 days before experiments and followed by the postoperative testing of reversal learning for 7 days. A, Escape latency during the experiments. B, Percentage of time spent in the target quadrant during the experiments. C, Swimming path length during the experiments. D, Swimming speed during the experiments. E, The probe trial test at day 3 and day 7 after surgery. F, Representative swim paths for S-R and S-P group at day 3 after surgery. The above data are shown as mean ± SEM; n = 10 for training; n = 8 for testing. *P<0.05 v.s. S-S group, #*P*<0.05 v.s. S-R group. S-S, sham surgery plus placebo; S-P, surgery plus placebo; S-R, surgery plus RNase.

Assessment of hippocampal-dependent learning and memory by MWM in all treatment groups at day 3 postoperatively demonstrated a statistically significant reduction in memory retrieval in S-P group compared to the S-S group, as evidenced by escape latency, path length, and percentage of time spent in target quadrant respectively (**Fig 2A–2C**), suggesting that unilateral nephrectomy induced a significant cognitive impairment in aged mice, therefore emulating POCD. The S-P group had similar swimming speed as that of the S-S group either pre or postoperatively (**Fig 2D**), implying that surgical operation per se had no impact on exercise capacity of the mice. Importantly, RNase treatment led to significantly enhanced cognitive function, compared with untreated surgical group, as demonstrated by escape latency (23.01 ± 5.15 vs. 43.67 ± 7.23, #*P*< 0.05) (**Fig 2A**), path length (333.20 ± 60.60 vs. 636.33 ± 89.72, #*P*<0.05) (**Fig 2B**), and percentage time spent in target quadrant (27.08 ± 2.84% vs. 24.21 ± 1.33%, #*P*<0.05) (**Fig 2C**), indicating that RNase treatment can attenuate POCD induced by unilateral nephrectomy in aged mice. Likewise, there was no difference in swimming speed either pre or postoperatively between S-R group and S-P group (**Fig 2D**). Additionally, a probe test was conducted at day 3 and day 7 after surgery to test whether the mice had learned and could recall the spatial location of the hidden platform. The percentage of time spent in the quadrant with the previously located hidden platform was significantly less in the S-P group undergoing probe tests (29.00 ± 1.88% and 36.12 ± 0.80%) when compared with the S-S group (39.48 ± 3.06% and 42.00 ± 2.11%) on day 3 and day 7 after surgery, respectively (**Fig 2E**, *P*<0.05). The S-R group undergoing probe tests had a significantly higher percentage of time spent in the target quadrant compared to that of the S-P group at day 3 after surgery (37.66 ± 2.28% vs. 29.00 ± 1.88%, #*P*< 0.05) (**Fig 2E**). Additionally, RNase treatment had no impact on the sham group (data not shown). Taken together, the above results revealed that unilateral nephrectomy induced a significant cognitive impairment, which could be attenuated by perioperative RNase treatment.

### Effects of RNase Treatment on Physiologic Parameters and Mortality

The comparison of the arterial blood gas parameters among groups (S-S, S-P, S-R and naive) is summarized in **Table 1**. Physiologic parameters, such as pH, blood gas (pCO2, pO2), electrolytes (cCl-, cCa++, cK+, cNa+), metabolites (cLac) and oximetry (ctHb, sO2), had no significant difference among groups (S-S, S-P, and S-R) immediately following surgery when compared with that in the naive controls (**Table 1**). Also, mice undergoing RNase treatment all survived on the observation days after surgery. Only one mouse from the S-S group in the experiment of MWM testing died at day 1 after surgery (data not shown). These results indicate that perioperative RNase treatment used in our study did not have any impact on the physiology parameters and mortality.

**Table 1:** Analysis of arterial blood gas parameters.

	pH	pCO2(mmHg)	pO2 (mmHg)	ctHb(g/dL)	sO2(%)	cK	cNa^+^	cCa2^+^	cCl^-^	cLac
						(mmol/L)				
**Naïve group**	7.38±0.04	43.83±2.17	94.97±1.17	10.97±1.95	90.63±5.00	5.30±0.10	155.67±3.48	1.24±0.13	122.00±0.58	1.77±0.48
**S-S group**	7.36±0.03	44.13±2.64	100.17±7.49	11.67±0.34	88.00±1.17	5.30±0.17	162.00±4.62	1.14±0.03	124.33±1.76	1.73±0.12
**S-P group**	7.36±0.01	46.43±1.68	99.80±5.69	13.97±0.72	86.70±2.20	5.43±0.24	152.33±3.84	1.16±0.05	121.67±0.67	1.03±0.19
**S-R group**	7.36±0.02	45.30±1.29	93.57±1.17	11.17±0.62	88.07±0.70	5.57±0.13	156.33±1.86	1.17±0.05	123.00±0.58	1.83±0.18

Data are shown as mean ± SEM (n = 3). There are no statistically significant differences for all the physiology parameters among the four groups. S-S, sham surgery plus placebo; S-P, surgery plus placebo; S-R, surgery plus RNase.

### Effect of RNase Treatment on DNA-Free Total Serum RNA

**Fig 3:**
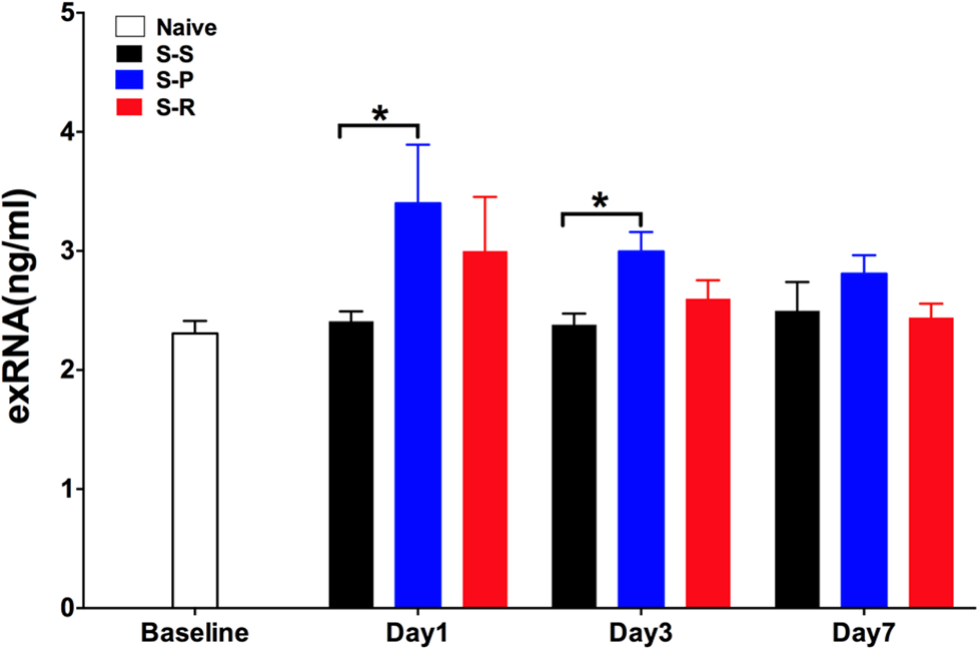
Effect of RNase administration on total serum RNA after unilateral nephrectomy in aged mice. The concentration of total serum RNA at day 1, day 3 and day 7 after surgery in aged mice from all three groups and the naïve control group were measured. Data are presented as mean ± SEM (n = 5 per group; n = 6 for naïve control group). *P<0.05 vs. S-S group. S-S, sham surgery plus placebo; S-P, surgery plus placebo; S-R, surgery plus RNase.

As one of the important DAMPs, exRNA, such as ribosomal RNA, transfer RNA, and messenger RNA, may be released from damaged cells during surgical injury. Therefore, we measured the total serum RNA levels at day 1, day 3 and day 7 after surgery in the S-S, S-P, and S-R group, in order to evaluate the functional role of exRNA in nephrectomy induced cognitive dysfunction. We have found total serum RNA was significantly increased in the S-P group compared with that of the S-S group at day 1 (3.40± 0.49 v.s. 2.41 ± 0.08, *P<0.05, n = 5) and day 3 (3.00 ± 0.16 v.s. 2.38 ± 0.09, *P<0.05, n = 5) after surgery, whereas there was no difference between S-S group and Naïve group (n = 6 for naïve group) (**Fig 3**). Notably, total serum RNA level had a decreasing trend in the S-R group at day 3 (*P* = 0.15) and day 7 (*P* = 0.09) postoperatively (n = 5) (**Fig 3**). This suggests that perioperative administration of RNase, the counterpart of exRNA, may reduce the serum levels of RNA. Taken together, these data suggest that the protective effect of RNase treatment on the unilateral nephrectomy-induced cognitive dysfunction might be associated with the reduction of exRNA released after surgical injury.

### RNase Decreased Cytokines Expression in the Serum and Hippocampus after Unilateral Nephrectomy

**Fig 4:**
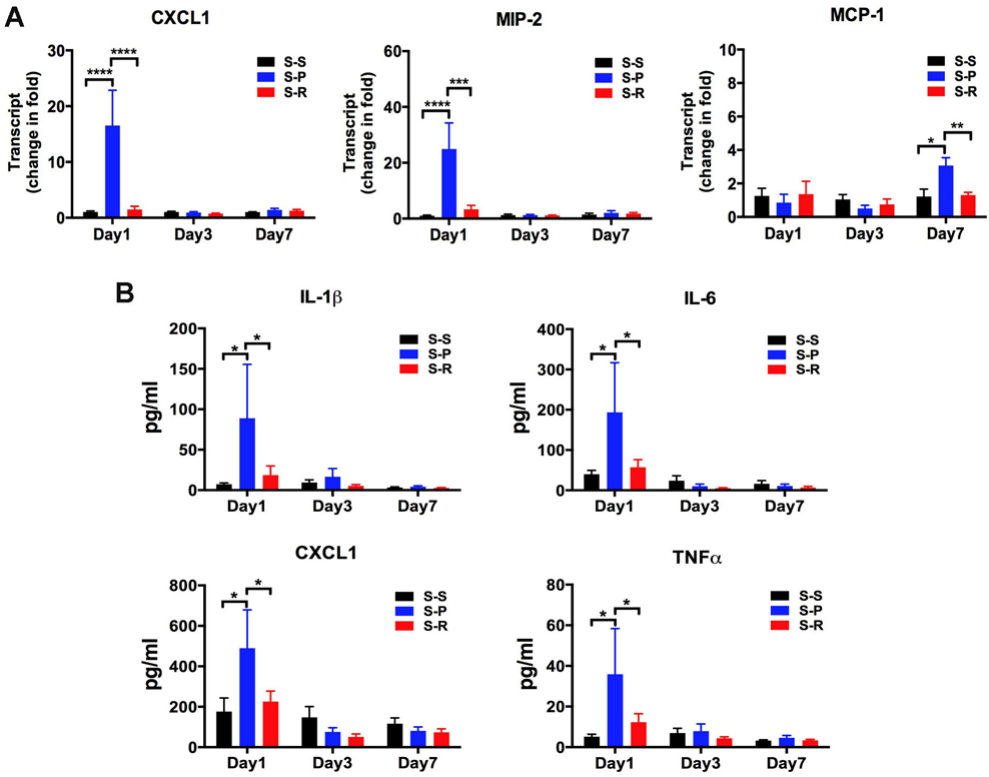
RNase treatment decreased cytokine expression in the hippocampus and serum after unilateral nephrectomy in aged mice. A, Hippocampal cytokine mRNA. At day 1, day 3 and day 7 after surgery, cytokine mRNA levels were measured using qRT-PCR. B, Serum cytokine protein expression. At day 1, day 3 and day 7 after surgery, cytokine proteins were assayed using Luminex. Data are presented as mean ± SEM (panel A, n = 4 per group; panel B, n = 6 per group). *P<0.05, **P<0.01, ***P<0.001, ****P<0.0001. CXCL1, chemokine (C-X-C mortif) ligand 1; MIP-2, macrophage inflammatory protein-2; MCP-1, monocyte chemotactic protein-1; IL, interleukin; TNF, tumor necrosis factor; S-S, sham surgery plus placebo; S-P, surgery plus placebo; S-R, surgery plus RNase.

Unilateral nephrectomy dramatically increased mRNA expression of CXCL1 and MIP-2 in the hippocampus at day 1 in aged mice compared with the sham control. This increase was significantly reduced by perioperative treatment with 3 doses of RNase (**Fig 4A,** ***P<0.001, ****P<0.0001). For MCP-1 the increase in the S-P group reached significance at day 7 after surgery and was also attenuated by RNase treatment (**Fig 4A,** *P<0.05, **P<0.01). Serum cytokines protein expression including IL-1β, IL-6, KC, and TNFα were significantly increased in the S-P group compared with the sham control at day 1 after surgery, and the increase was also significantly decreased in the S-R group (**Fig 4B,** *P<0.05). There was no difference between the S-R group and the S-S group. These results indicate that neuroprotective effect of RNase may be mediated through reducing both peripheral and hippocampus inflammation.

### RNase Attenuated Unilateral Nephrectomy-induced Apoptosis in the Hippocampus

**Fig 5:**
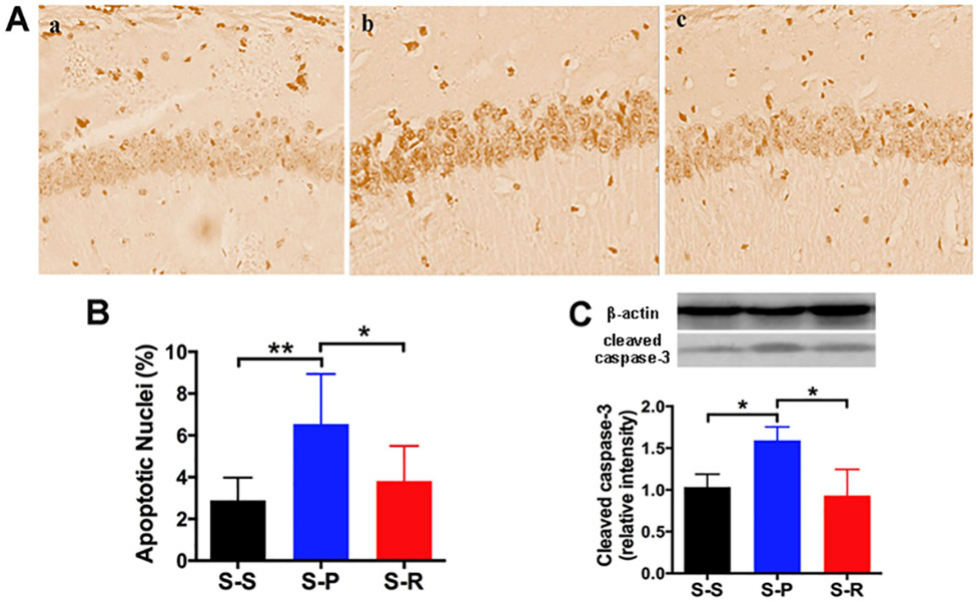
RNase treatment attenuated hippocampal apoptosis after unilateral nephrectomy in aged mice. A, TUNEL staining at day 1 after surgery. A-a, S-S group; A-b, S-P group; A-c, S-R group. B, Quantitation of TUNEL staining. C, Quantitation of relative intensity of cleaved caspase-3 bands and a representative blot at day 1 after surgery. Data are presented as mean ± SEM (panel B, n = 6 per group; panel C, n = 3 per group). *P<0.05, **P<0.01. S-S, sham surgery plus placebo; S-P, surgery plus placebo; S-R, surgery plus RNase.

The percentage of TUNEL-positive nuclei in the total nuclei of the hippocampal CA1 region was markedly increased in the S-P group, compared with the S-S group at day 1 after surgery. The increase was significantly inhibited by administration of RNase (**Fig 5A and 5B,** *P<0.05, **P<0.01). Protein expression of cleaved caspase 3 in the hippocampus was significantly increased at day 1 in the S-P group compared with the sham control, and RNase treatment significantly reduced this increase (**Fig 5C,** *P<0.05). There was no difference between the S-S group and the S-R group. Besides, for both TUNEL staining and cleaved caspase 3, no differences were observed among the three groups (data not shown). The above data suggest that attenuation of hippocampal apoptosis in the early stage after surgery may also be involved in the neuroprotection provided by RNase treatment.

### Kidney Function after Experiments

In terms of the postoperative kidney function, reflected by the serum levels of CREA and BUN, there were no significant changes among groups (S-S, S-P, S-R) at day 1, day 3 and day 7, when compared to the naive controls (**Table 2**).

**Table 2:** Kidney function after experiments.

		CREA(μmol/L)	BUN(mmol/L)
**Naïve group**		38.78±1.03	7.26±0.37
**S-S group**	**Day 1**	39.57±0.41	10.76±1.40
	**Day 3**	39.60±0.81	9.52±0.29
	**Day 7**	36.07±1.52	7.49±0.53
**S-P group**	**Day 1**	43.97±1.68	16.30±1.61
	**Day 3**	41.07±3.03	10.85±0.72
	**Day 7**	38.87±0.35	9.45±0.76
**S-R group**	**Day 1**	45.77±2.31	13.70±2.84
	**Day 3**	34.90±0.51	6.18±0.43
	**Day 7**	37.97±1.27	8.57±0.67

Data are shown as mean ± SEM (n = 3). There are no statistically significant differences among groups (S-S, S-P, and S-R) at day 1, day 3 and day 7 after surgery for both CREA and BUN when compared with the naïve control group. CREA, creatinine; BUN, blood urea nitrogen; S-S, sham surgery plus placebo; S-P, surgery plus placebo; S-R, surgery plus RNase.

## Discussion

In the present study, the effect of RNase treatment on cognitive function after unilateral nephrectomy was investigated in aged mice. Our data revealed that unilateral nephrectomy induced a significant cognitive impairment in aged mice at day 3 after surgery, while perioperative administration of RNase led to improved cognitive function at the corresponding time point. Moreover, RNase treatment decreased cytokine expression levels in the serum and hippocampus, and also inhibited hippocampal apoptosis induced by unilateral nephrectomy. In addition, significantly increased total serum RNA levels were detected in the surgery group compared with the sham control. However, there was a decreasing trend of total serum RNA levels in the RNase treated group at both day 3 and day 7, which implies a potential association between the increased exRNA and the initiation of POCD. Also, the protocol of RNase administration had no impact on the arterial blood gas parameters immediately following surgery, kidney function, and mortality rate at the observed days after surgical procedure.

Despite years of effort to find a possible treatment for patients with POCD, researchers still fail to fully understand the pathophysiology for the development of POCD [22]. Previously, various types of surgery, such as partial hepatolobectomy [23], splenectomy [14], and unilateral nephrectomy [24], have been reported for the establishment of an animal model for POCD. Increased neuroinflammation has been demonstrated to be the key mechanism for the development of POCD in animal models [7, 8]. However, clinical trials using anti-inflammatory drugs fail to show consistent benefits for patients with cognitive dysfunction [9]. Thus, we aimed to find a possible treatment that could work on the initiators at the very beginning stage involved in the initiation, amplification and perpetuation of neural inflammation or apoptosis. Previous studies have shown increased concentration of protein levels of systemic S-100β and HMGB-1, and their pivotal role in surgery-induced cognitive deficits [14, 15]. It is known that, both S-100β and HMGB-1 are the important DAMPs molecules, which might be generated and released during adverse conditions, mediating responses of the immune system in the noninfectious inflammatory processes [13]. As one of the important DAMPs molecules, exRNA, is able to remain in circulation after being released from necrotic cells, because it is protected by exsome, binding-proteins from the digestion of RNase existing within our circulatory system [25]. Previously, released exRNA was reported to be involved in the pathogenesis of myocardial I/R injury [16], atherosclerotic plaque formation [17], and focal cerebral ischemia [18]. In the present study, we have also detected significantly increased total serum RNA levels at day 1, day 3 and day 7 after unilateral nephrectomy compared with the sham control. Increased serum levels of exRNA in the mice model of POCD may indicate a possible association between exRNA and the cognitive injury after surgery.

RNase, the counter part of RNA, was mostly reported as a promising novel anticancer drug target [26]. Studies have demonstrated that brain edema and infarction induced by cerebral ischemia reperfusion were reduced by RNase treatment [18, 19]. Also, RNase treatment led to significantly decreased myocardial infarction induced by myocardial I/R injury [16]. However, to the best of our knowledge, the effect of RNase treatment on the development of POCD has not been explored. Thus, we proposed that exRNA may play in an important role in the development of POCD, and RNase A, through digesting the exRNA, may help to improve cognitive impairment induced by surgical injury in aged mice. In our present study, we, for the first time, demonstrate that RNase A treatment leads to cognitive improvement after unilateral nephrectomy in aged mice. The protocol of perioperative RNase A administration we used showed a good tolerability in aged mice, as it had no impact on arterial blood gas parameters, serum levels of CREA or BUN and mortality rate after the surgical procedure. However, we did not find a significant reduction of total serum RNA levels after RNase treatment, but only found a decreasing trend at day 1, day 3 and day 7 after surgery. The reason that we failed to obtain a significant difference of total serum exRNA may be due to the fact that RNase A degrades ssRNA efficiently, but not other exRNA, such as rRNA, mRNA and miRNA. Although our data has revealed the protective effect of RNase administration against the development of POCD, further study is required to confirm whether this protection is associated with the degradation of serum exRNA. Additionally, confirmation of which specific exRNA is involved in this process would then be warranted.

Molecular mechanisms for the neuroprotection provided by RNase may be related to the reduction of both peripheral and central inflammation, and hippocampus apoptosis. In our study, serum protein levels of IL-1β, IL-6, CXCL-1 and TNF-α were significantly increased at day 1 after surgery, which is similar to the findings of previous research [7]. Notably, RNase treatment markedly suppressed the increased protein levels of these cytokines. To our surprise, we only found RNase treatment significantly reduced the mRNA levels of chemokines, namely, CXCL1, MIP-2 and MCP-1 in the hippocampus at day 1 or day 7 after surgery, but failed to detect differences for IL-1β, IL-6, and TNF-α (data not shown). Also, cleaved caspase-3 and TUNEL-positive nuclei in the hippocampal CA1 region were observed to be significantly diminished after RNase treatment at day 1 after surgery. Similarly, a previous study by Terrando et al. [7] showed an early increased level of systemic pro-inflammatory cytokines, like TNF-α, 30 min after surgery in aged mice subjected to an open tibial fracture. Meanwhile, Rudolph et al. [27], in a clinical study, found that increased levels of chemokine in the early stages after surgery was associated with patients developing POCD. The reason that we only observed the changes of chemokines, mRNA levels, and apoptosis in the hippocampus at 1 day may be because the mice model we used was a relatively mild-to-moderate injury model. The innate recovery process of the mice, automatically activated after the surgical injury, may lead to the attenuation of exRNA, and therefore the later reduction of chemokine levels. Moreover, Kong et al. [21] has also demonstrated an early increased apoptosis (4 hours after isoflurane exposure) in the hippocampus of aged rats with cognitive decline. Although the exact mechanism behind these results remains elusive, we believe that released exRNA may induce the early-stage increased hippocampal chemokines and apoptosis by acting on the peripheral or central DAMPs receptor. This may then trigger a cytokine and/or apoptotic cascade contributing to the development of POCD. Admittedly, our current study still cannot rule out the possibility that peripheral cytokines may pass through blood brain barrier to act as the initiators of neural injury after surgery.

Our study certainly has some limitations. We treated the mice with RNase A, one of the pancreatic-type ribonucleases that is of high efficiency for the degradation of ssRNA substrates. However, it remains elusive as to what the specific kind of exRNA and how complete the digestion would be affected by the systemically administered RNase A *in vivo*, since certain microvesicles or RNA-binding proteins associated RNA have been found to be protected from digestion. Also, RNase A has a small molecular weight (17 kDa) and has been found to have a short (about several minutes) plasma half-life, with a rapid clearance primarily via kidney [28]. It could be more effective and lasting for the digestion of exRNA if an osmotic capsule could be used to continuously administer RNase A. Further, our results indicate a possible causal relationship between the increased serum exRNA and the initiation of POCD. Further study is required to detect the exRNA level in the cerebral spinal fluid to further support the association between increased exRNA and cognitive impairment. Finally, although our work has indicated that reduction of inflammation and apoptosis might be the underlying mechanisms that are involved in the cognitive protection effects of RNase, the detailed signaling mechanism remains to be defined.

## Conclusion

In summary, the results of our study revealed that RNase treatment may attenuate unilateral nephrectomy-induced cognitive impairment in aged mice. Perioperative RNase administration might cause a trending decrease of exRNA in the serum, but had no impact on physiological parameters and mortality. RNase treatment may exhibit cognitive protective effect by reducing peripheral inflammation, hippocampal inflammation, and apoptosis. The current study may help to expand the knowledge base for a novel therapy for POCD, and RNase may be a promising candidate for the improvement of cognitive function after surgery. Further studies are still needed to fully characterize such a phenomenon and elucidate the detailed underlying mechanisms.
